# Pleural fluid microbiota as a biomarker for malignancy and prognosis

**DOI:** 10.1038/s41598-023-29001-4

**Published:** 2023-02-08

**Authors:** Benjamin Kwok, Benjamin G. Wu, Ibrahim F. Kocak, Imran Sulaiman, Rosemary Schluger, Yonghua Li, Raheel Anwer, Chandra Goparaju, Daniel J. Ryan, Marla Sagatelian, Matthew S. Dreier, Vivek Murthy, Samaan Rafeq, Gaetane C. Michaud, Daniel H. Sterman, Jamie L. Bessich, Harvey I. Pass, Leopoldo N. Segal, Jun-Chieh J. Tsay

**Affiliations:** 1 Division of Pulmonary, Critical Care, and Sleep Medicine, New York University Grossman School of Medicine, 462 First Avenue 7N21, New York, NY 10016 USA; 2 Division of Pulmonary and Critical Care Medicine, Veterans Affairs New York Harbor Healthcare System, New York, NY USA; 3 Department of Respiratory Medicine, Royal College of Surgeons in Ireland, Dublin, Ireland; 4 Department of Respiratory Medicine, Beaumont Hospital, Dublin, Ireland; 5 Department of Cardiothoracic Surgery, New York University Grossman School of Medicine, New York, NY USA; 6 School of Medicine, Medical University of South Carolina, Charleston, SC USA; 7 New York University Grossman School of Medicine, New York, NY USA; 8 Division of Pulmonary, Critical Care, and Sleep Medicine, University of South Florida Health, Tampa, FL USA

**Keywords:** Cancer microenvironment, Prognostic markers, Non-small-cell lung cancer, Microbiome

## Abstract

Malignant pleural effusions (MPE) complicate malignancies and portend worse outcomes. MPE is comprised of various components, including immune cells, cancer cells, and cell-free DNA/RNA. There have been investigations into using these components to diagnose and prognosticate MPE. We hypothesize that the microbiome of MPE is unique and may be associated with diagnosis and prognosis. We compared the microbiota of MPE against microbiota of pleural effusions from non-malignant and paramalignant states. We collected a total of 165 pleural fluid samples from 165 subjects; Benign (*n* = 16), Paramalignant (*n* = 21), MPE-Lung (*n* = 57), MPE-Other (*n* = 22), and Mesothelioma (*n* = 49). We performed high throughput 16S rRNA gene sequencing on pleural fluid samples and controls. We showed that there are compositional differences among pleural effusions related to non-malignant, paramalignant, and malignant disease. Furthermore, we showed differential enrichment of bacterial taxa within MPE depending on the site of primary malignancy. Pleural fluid of MPE-Lung and Mesothelioma were associated with enrichment with oral and gut bacteria that are commonly thought to be commensals, including *Rickettsiella*, *Ruminococcus*, *Enterococcus*, and *Lactobacillales*. Mortality in MPE-Lung is associated with enrichment in *Methylobacterium*, *Blattabacterium*, and *Deinococcus*. These observations lay the groundwork for future studies that explore host-microbiome interactions and their influence on carcinogenesis.

## Introduction

Malignancies that develop malignant pleural effusions (MPE) portend significantly poorer overall survival^1^. Early recognition and accurate diagnosis of MPE is paramount since its diagnosis changes staging of malignancy, therapeutic options, and prognosis. Malignant involvement of the pleural space is diagnosed by cytologic analysis of pleural fluid collected by minimally-invasive means, typically by needle thoracentesis; however, the sensitivity of cytologic analysis is less-than-ideal and repeat analyses typically has limited additive value to test performance^2^. This may lead to invasive procedures that are subject to sampling bias and place the patients at risk for complications that may negatively impact their quality of life.

The pleural space is not just a bystander in malignant disease states. In MPE, the pleural microenvironment may include circulating tumor cells, tumor-educated inflammatory cells, cell-free and circulating-tumor DNA, non-coding/microRNA, and tumor metabolites^3–13^. These components are theorized to play key roles in the development and progression of malignancy and MPE. There have been investigations into using components of pleural fluid as biomarkers to diagnose, detect mutations, and predict prognosis and response to therapies^5,8–10,14–17^.

The microbiome of pleural fluid has been described in certain infectious disease states including parapneumonic effusions and empyemas^18–21^. While associations between cancer and microbial DNA signatures in other human-derived samples have been identified^22,23^, the microbiome of MPE is not well characterized and may yield a novel biomarker^24^. In this study, we investigated whether the microbiome of MPE differs significantly from microbiota of paramalignant pleural effusions (cytological negative effusions associated with malignancies) and non-malignant pleural effusions. In addition, we sought to determine whether the microbial signatures are associated with prognosis and survival.

## Results

### Study cohort

A total of 165 subjects were included in this study. In addition to pleural fluid samples (165 samples), a total of 58 background samples (swabs of sterile surgical equipment and reagent control samples) and 21 skin samples (swabs collected after the skin was prepared with antiseptic solution as part of the pleural fluid collection procedure) were also collected. Table 1 shows the demographic and clinical characteristics of the included subjects. Pleural fluid samples were divided into five groups (see “Methods” for details): Benign (*n* = 16), Paramalignant (*n* = 21), MPE-Lung (*n* = 57), MPE-Other (*n* = 22), and Mesothelioma (*n* = 49). Subjects in the Benign group were more likely to have comorbidities including chronic heart failure (CHF), connective tissue disease (CTD), chronic kidney disease (CKD), and cirrhosis. Among the samples in the Benign group, half were transudative and half were exudative by Light’s criteria^25^ (Supplemental Table S1). A majority of subjects in the Paramalignant group was Caucasian (90%) and had non-small cell lung cancer (90%, NSCLC) (Table 1, Supplemental Table S2). All subjects from the MPE-Lung group had NSCLC, with a majority having adenocarcinoma (79%). Subjects in the MPE-Other group had predominantly adenocarcinomas (73%) and malignancies of gastrointestinal origin (36%). Within the Mesothelioma group, a majority of subjects was Caucasian (86%), male (69%), and had history of asbestos exposure (74%). Epithelial mesothelioma was the most common type of malignant pleural mesothelioma (88%).

**Table 1:** Subject demographics.

Characteristic	Total N = 165^1^	Benign N = 16^1^	Paramalignant N = 21^1^	MPE-Lung N = 57^1^	MPE-Other N = 22^1^	Mesothelioma N = 49^1^	p-value^2^
Age	71 [62, 79]	70 [56, 81]	74 [66, 82]	71 [61, 78]	72 [64, 80]	66 [60, 76]	0.3
Gender (male)	83 (50%)	9 (56%)	9 (43%)	23 (40%)	8 (36%)	34 (69%)	0.019^a,b^^,c^
BMI	24.9 [22.3, 27.5]	25.3 [23.7, 28.5]	25.8 [24.9, 27.9]	24.4 [21.8, 27.1]	24.4 [22.7, 26.5]	25.0 [21.9, 27.6]	0.3
Race							0.02
Caucasian	122 (74%)	11 (69%)	19 (90%)	35 (61%)	15 (68%)	42 (86%)	< 0.001^a,b^^,d,e,f,i^
Hispanic	5 (3.0%)	1 (6.2%)	0 (0%)	2 (3.5%)	0 (0%)	2 (4.1%)	
African-American	11 (6.7%)	1 (6.2%)	1 (4.8%)	3 (5.3%)	4 (18%)	2 (4.1%)	
Asian	11 (6.7%)	0 (0%)	1 (4.8%)	10 (18%)	0 (0%)	0 (0%)	
Other	16 (9.7%)	3 (19%)	0 (0%)	7 (12%)	3 (14%)	3 (6.1%)	
Smoker	92 (56%)	8 (50%)	15 (71%)	36 (63%)	9 (41%)	24 (49%)	0.2
Pack-years	20 [8.6, 46.5]	30 [9, 40.5]	47 [15, 67]	27 [10, 45]	5 [3, 26]	15 [10, 23.8]	0.15
Asbestos exposure	33 (22%)	0 (0%)	1 (5.0%)	1 (2.0%)	0 (0%)	31 (74%)	< 0.001
Comorbidities							
Hyperlipidemia	74 (48%)	9 (56%)	11 (55%)	19 (39%)	10 (45%)	25 (53%)	0.5
Hypertension	85 (55%)	10 (62%)	12 (60%)	25 (51%)	15 (68%)	23 (49%)	0.5
Heart failure	24 (16%)	7 (47%)	5 (25%)	6 (12%)	2 (9.1%)	4 (8.5%)	0.009^d,e^^,f,g^
CAD	33 (21%)	5 (31%)	4 (20%)	11 (22%)	2 (9.1%)	11 (23%)	0.5
CVA	9 (5.8%)	1 (6.2%)	2 (10%)	5 (10%)	0 (0%)	1 (2.1%)	0.3
Diabetes mellitus	31 (20%)	3 (20%)	5 (25%)	8 (16%)	4 (18%)	11 (23%)	0.9
Asthma	8 (5.2%)	0 (0%)	0 (0%)	2 (4.1%)	2 (9.1%)	4 (8.5%)	0.5
COPD	25 (16%)	2 (12%)	7 (35%)	8 (16%)	3 (14%)	5 (11%)	0.2
CTD	15 (9.7%)	7 (44%)	1 (5.0%)	2 (4.1%)	2 (9.1%)	3 (6.4%)	0.001^d,e^^,f,g,h^
CKD	17 (11%)	7 (44%)	1 (5.0%)	4 (8.2%)	2 (9.1%)	3 (6.4%)	0.005^d,e^^,f,g,h^
Cirrhosis	4 (2.6%)	3 (19%)	0 (0%)	0 (0%)	0 (0%)	1 (2.1%)	0.007^d,e^^,f,g,h,f,i^
HIV infection	0 (0%)	0 (0%)	0 (0%)	0 (0%)	0 (0%)	0 (0%)	> 0.9
GERD	49 (32%)	4 (25%)	7 (35%)	16 (33%)	9 (41%)	13 (28%)	0.8

*CAD* coronary artery disease, *CVA* cerebrovascular accident, *COPD* chronic obstructive pulmonary disease, *CTD* connective tissue disease, *CKD* chronic kidney disease, *HIV* human immunodeficiency disease infection, *GERD* gastroesophageal reflux disease.

^1^Median [IQR]; n (%).

^2^Kruskal–Wallis rank sum test; Pearson's Chi-squared test; Wilcox rank sum test.

^a^Chi-square or Wilcox Rank Sum test between groups Paramalignant and Mesothelioma had a p-value < 0.05.

^b^Chi-square or Wilcox Rank Sum test between groups MPE-Lung and Mesothelioma had a p-value < 0.05.

^c^Chi-square or Wilcox Rank Sum test between groups MPE-Other and Mesothelioma had a p-value < 0.05.

^d^Chi-square or Wilcox Rank Sum test between groups Benign and MPE-Lung had a p-value < 0.05.

^e^Chi-square or Wilcox Rank Sum test between groups Benign and Mesothelioma had a p-value < 0.05.

^f^Chi-square or Wilcox Rank Sum test between groups Paramalignant and MPE-Lung had a p-value < 0.05.

^g^Chi-square or Wilcox Rank Sum test between groups Benign and MPE-Other had a p-value < 0.05.

^h^Chi-square or Wilcox Rank Sum test between groups Benign and Paramalignant had a p-value < 0.05.

^i^Chi-square or Wilcox Rank Sum test between groups MPE-Lung and MPE-Other had a p-value < 0.05.

^f^Chi-square or Wilcox Rank Sum test between groups Paramalignant and MPE-Other had a p-value < 0.05.

### The microbiota in pleural fluid is distinct from that found in background and skin samples

We evaluated the characteristics of the microbiota in pleural fluid compared to those of background and skin samples, which we considered as potential sources of microbial genomic contamination to the pleural fluid samples. Median sequence depth was 21,138 [IQR 12,489, 29,502] counts per sample and sequence depths were comparable among different sample types (Supplemental Fig. S1). A total of 11,250 taxa were identified. After filtering for taxa not seen more than five times in 1% of samples and agglomerating at the genus-level, a total of 551 taxa remained for downstream analysis. Bacterial load, as assessed by digital droplet PCR (ddPCR), was similar between background and skin swabs; there was a greater range of bacterial load in pooled pleural fluid than in background and skin samples (Supplemental Fig. S2). Alpha diversity (Shannon index) was statistically different between background and skin samples. Beta diversity (Bray–Curtis dissimilarity index) showed clear compositional differences among background, skin, and pleural fluid samples (Supplemental Fig. S2, PERMANOVA *p* < 0.001).

### The microbiota in pleural fluid varies between disease states

**Figure 1:**
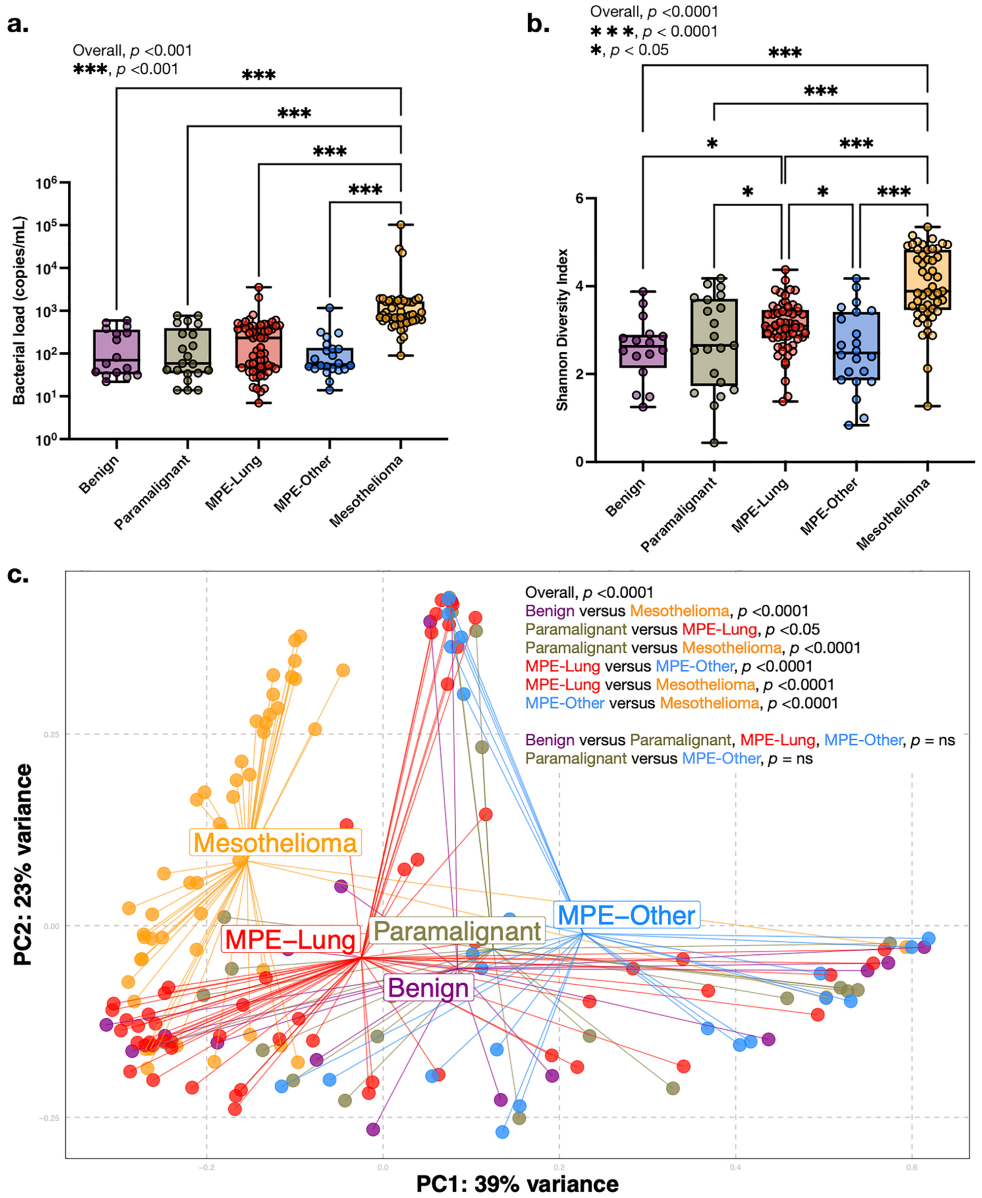
Microbial compositional differences between groups of pleural fluid. (**a**) Bacterial load (copies/mL) by ddPCR. *p*-values by Kruskal–Wallis rank sum test; individual comparisons by Wilcoxon rank sum tests with Benjamini–Hochberg adjustment for multiple comparisons. (**b**) Alpha diversity (Shannon diversity). *p*-values by Kruskal–Wallis rank sum test. Individual comparisons by Wilcoxon rank sum tests with Benjamini–Hochberg adjustment for multiple comparisons. (**c**) Beta diversity (Bray–Curtis dissimilarity index). *p*-values by PERMANOVA. *ns* not significant.

We evaluated microbiota differences in pleural fluid samples among different histopathologic groups. Bacterial load was greater in the Mesothelioma group compared to the other groups (Fig. 1a). Alpha diversity (Shannon index) was greater among the MPE-Lung and Mesothelioma groups as compared with the other pleural fluid groups (Fig. 1b). Beta diversity revealed differences in composition among the five groups (*p* < 0.05, Fig. 1c).

**Figure 2:**
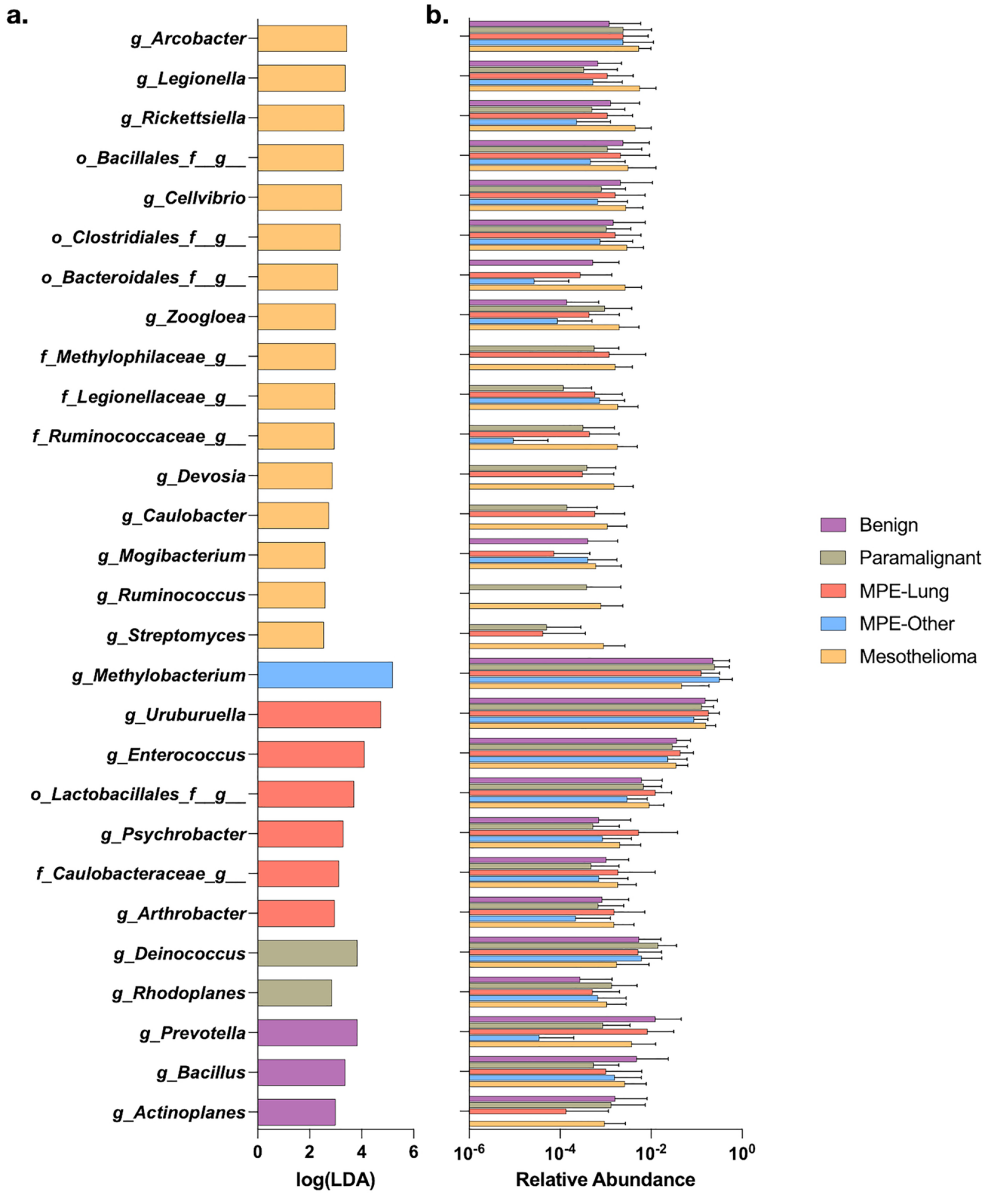
Taxonomic differences between groups of pleural fluid. (**a**) Identification of taxa enriched in group of pleural fluid by linear discriminant analysis effect size (LEfSe). (**b**) Mean relative abundance for each taxa identified as enriched by LEfSe.

To explore taxonomic differences among the different histopathologic groups, we performed linear discriminant analysis effect size (Fig. 2, LEfSe). Among taxa not identified as potential contaminant, pleural fluid from the Benign group was enriched with *Prevotella* and *Bacillus* while pleural fluid from the Paramalignant group was enriched with *Deinococcus*. Pleural fluid from the MPE-Lung group was enriched with *Enterococcus*, *Lactobacillales*, *Psychrobacter*, and *Caulobacteraceae* while the MPE-Other group was enriched in *Methylobacterium* and the Mesothelioma group was enriched with *Legionella*, *Rickettsiella*, and *Ruminococcus* (see Supplemental Table S3 for full list of results). These data support the hypothesis that different microbial signatures in pleural fluid are associated with distinct disease states.

We then applied Dirichlet multinomial mixtures (DMM) modeling on all pleural fluid samples to identify distinct profiles of the pleural microbiome. DMM identified three clusters that showed distinct alpha and beta diversities (Supplemental Fig. S2). Supplemental Table S4 shows the distribution of samples based on diagnosis across these three clusters. Benign, Paramalignant, and MPE-Other samples clustered mainly in Cluster 1; MPE-Lung samples were divided between Cluster 1 and Cluster 2; and Mesothelioma samples were mainly divided between Cluster 2 and Cluster 3. There were differences in demographics and clinical comorbidities amongst the three DMM clusters, but these were expected given the distribution of diagnoses (Supplemental Tables S4 and S5). LEfSe analyses identified top differentially enriched taxa within each cluster (Supplemental Fig. S4, Supplemental Table S6). The taxa identified in each cluster were similar to those identified based on the histopathological groups. For instance, the taxa *Deinococcus* was enriched in Cluster 1, in agreement with its high prevalence in Benign, Paramalignant and MPE-Other samples; *Enterococcus* was enriched in Cluster 2, also in agreement with its high prevalence in MPE-Lung and Mesothelioma samples. *Ruminococcus*, *Prevotella*, and *Rickettsiella* were enriched in Cluster 3, in agreement with their high relative abundance in MPE-Other and Mesothelioma samples.

### Survival is associated with differences in microbiome composition

**Figure 3:**
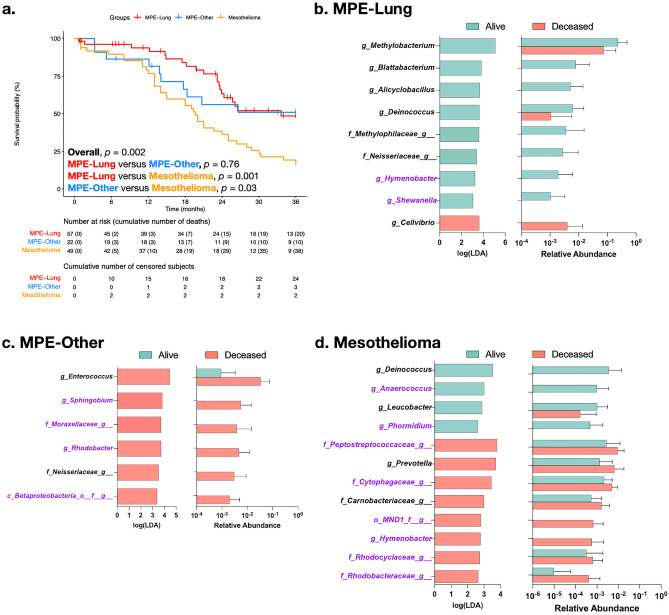
Taxonomic differences based on survival analysis. (**a**) Kaplan–Meier curves for groups of malignant pleural effusions. (**b**) Differential enrichment analysis by LEfSe for survival of subjects in the MPE-Lung group at time of median survival (33.6 months), MPE-Other group at time of median survival (36 months), and Mesothelioma group at time of median survival (19.6 months). Taxa colored in purple are potential contaminants.

Since the microbiome has been associated with clinical prognosis in other respiratory conditions^26^, we evaluated for associations between the microbiota in pleural fluid and clinical prognosis among the malignant groups (MPE-Lung, MPE-Other, and Mesothelioma, Supplemental Fig. S5). Figure 3a showed an overall Kaplan–Meier analyses for the three groups, noting that the median survival time was 33.6 months for the MPE-Lung group, 36 months for the MPE-Other group, and 19.6 months for the Mesothelioma group.

For each disease group, subjects were divided into those who survived beyond the median survival time and those who were deceased before the median survival time. We excluded subjects with follow-up less than the median survival time. Therefore, we analyzed 34/57 (59.6%), 19/22 (86%), and 54/54 (100%) subjects from the MPE-Lung, MPE-Other, and Mesothelioma groups, respectively. In the MPE-Lung group, 15 subjects were labeled as alive and 19 as deceased. In the MPE-Other group, 9 subjects were labeled as alive and 10 as deceased; in the Mesothelioma group, 24 subjects were labeled as alive and 20 as deceased.

We compared bacterial concentration and microbiome metrics between those labeled as alive and deceased in the histopathologic groups and found no statistically significant differences (Supplemental Fig. S6). We used LEfSe to explore taxonomic differences between these groups (Fig. 3b–d). For subjects in the MPE-Lung group, survival beyond the median survival time was associated with enrichment with *Methylobacterium*, *Blattabacterium*, and *Deinococcus*, whereas early mortality was associated with enrichment with *Cellvibrio*. For subjects in the MPE-Other group, early mortality was associated with the enrichment of *Enterococcus* and *Neisseriaceae* in the pleural space. Finally, for subjects in the Mesothelioma group, survival was again associated with again the enrichment of *Deinococcus*, whereas early mortality was associated with the enrichment of *Prevotella*.

**Figure 4:**
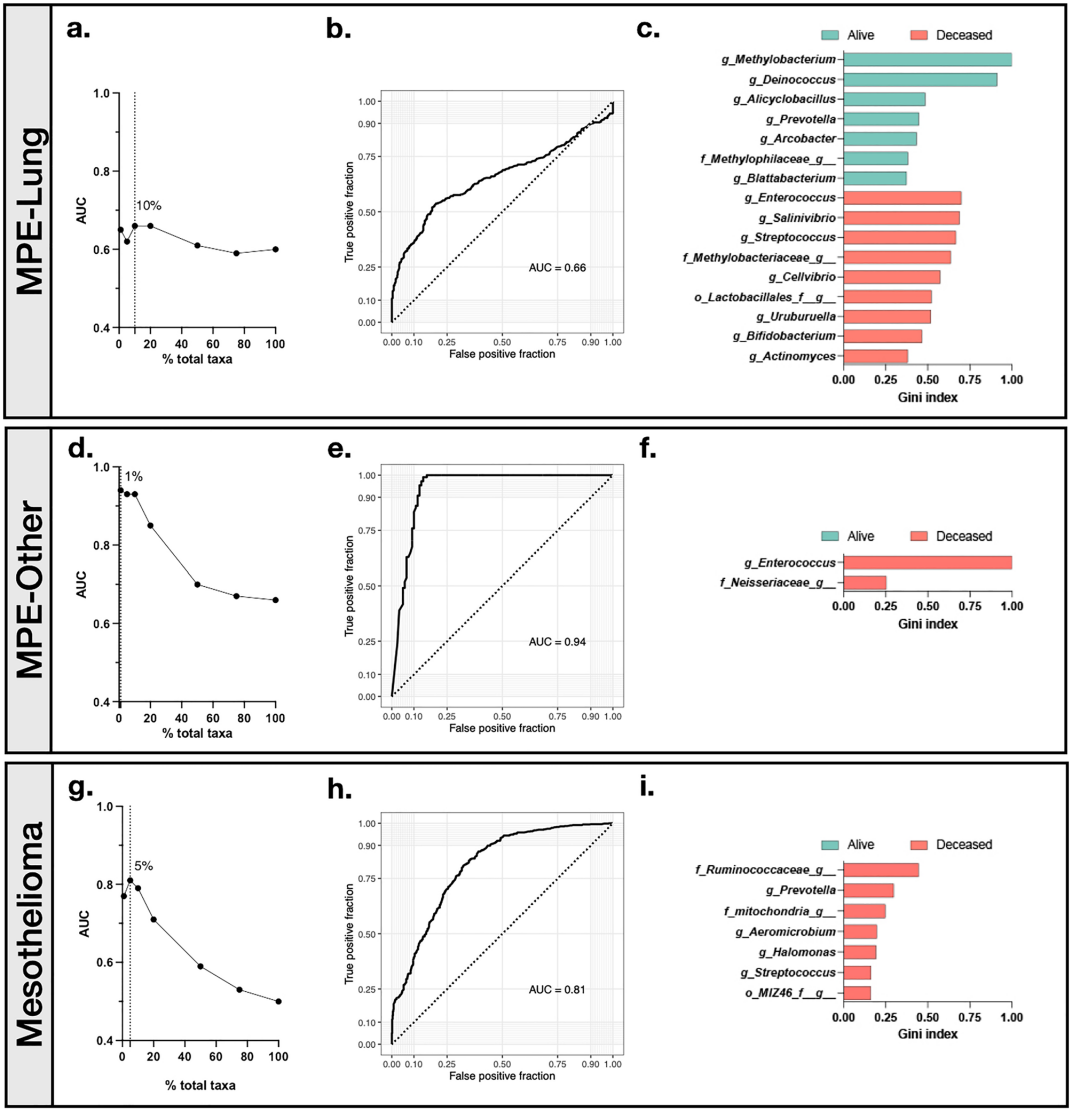
Random forest classifiers to predict mortality at median time of survival. (**a**) Area under the curve (AUC) of receiver operator curves (ROC) based on random forest identification of taxonomic classifiers predicting mortality in MPE-Lung median mortality (33.6 months) using the top 1, 5, 10, 20, 50, 75, and 100% of total discriminant taxa based on Gini values (*n* = 343). (**b**) ROC for the best-fit random forest classifier in MPE-Lung. (**c**) Taxa with greatest Gini Index from the random forest classifier with the greatest AUC (10% of taxa). (**d**) AUC of ROC based on random forest identification of taxonomic classifiers predicting mortality in MPE-Other median mortality (36 months) using the top 1, 5, 10, 20, 50, 75, and 100% of total discriminant taxa based on Gini values (*n* = 254). (**e**) ROC for the best-fit random forest classifier in MPE-Other. (**f**) Taxa with greatest Gini Index from the random forest classifier with the greatest AUC (1% of taxa). The taxa colored in purple is a potential contaminant. (**g**) AUC of ROC based on random forest identification of taxonomic classifiers predicting median mortality in Mesothelioma (19.6 months) using the top 1, 5, 10, 20, 50, 75, and 100% of total discriminant taxa based on Gini values (*n* = 503). (**h**) ROC for the best-fit random forest classifier in Mesothelioma. (**i**) Taxa with greatest Gini Index from the random forest classifier with the greatest AUC (5% of taxa). Potential contaminants are not shown.

We next generated random forest classifiers for MPE-Lung, MPE-Other, and Mesothelioma groups to best predict worst mortality based on median survival time using taxonomic features. Once key discerning taxonomic features were identified (based on Gini index) for each classifier, we tested the classifiers’ abilities to predict mortality based on calculated area under the curve (AUC) of receiver operating curves (ROC) using top 1, 5, 10, 20, 50, 75, and 100% of total discriminant taxa. For MPE-Lung, the AUC ranged from 0.59 to 0.66, with the best AUC identified with the use of top 10% taxa (Fig. 4a–c, Supplemental Table S7). As expected, several of these taxa were also identified in the LEfSe analyses, including *Methylobacterium*, *Deinococcus*, and *Cellvibrio*. For MPE-Other, the AUC ranged from 0.66 to 0.94, with the best AUC identified with the use of top 1% taxa (Fig. 4d–f, Supplemental Table S8). The top taxa identified by random forest were also concordant with LEfSe analyses (*Enterococcus*, *Neisseriaceae*, and *Rhodobacter*). For Mesothelioma, AUC ranged from 0.5 to 0.81, with the best AUC identified with 5% of available taxa (Fig. 4g–i, Supplemental Table S9). Top taxa identified by random forest and by LEfSe that were associated with prognosis in Mesothelioma were, again, similar. In this exploratory cohort, a select set of taxonomic signatures can perform with relatively high predictive power for prognosis. A validation cohort is required to accurately assess these classifiers’ potential value as biomarkers for prognosis.

## Discussion

The pleural space in malignancy is a complex compartment composed of not only immune and cancer cells, but also cell-free DNA/RNA. Furthermore, the components within that space are in constant equilibrium with other compartments of the human body and the systemic circulation^8–10,14,15^. Using 16S rRNA gene sequencing, we demonstrated that the microbiota of malignant pleural effusions related to NSCLC and mesothelioma were distinct from that of non-malignant and Paramalignant etiologies. Moreover, we identified microbial signatures associated with poor prognosis among thoracic malignant disease, which suggest that the microbiota in pleural fluid could potentially serve as a novel biomarker.

The microbiota in fluid from Benign and Paramalignant groups have greatest similarity, as suggested by similar diversity indices and their co-clustering in DMM modeling, implying a common etiology that is probably related to extravasation due to oncotic pressure and vascular system permeability. In contrast, amongst malignant pleural effusions, there are differences in bacterial diversity and composition depending on the malignant condition. Our analyses identified that the microbiota of Mesothelioma and MPE-Lung were enriched with bacteria commonly seen in the upper gastrointestinal (GI) tract, including *Legionella*, *Rickettsiella*, *Ruminococcus*, *Cellvibrio*, *Enterococcus*, *Psychrobacter*, and *Caulobacteraceae*. Some of these taxa, such as *Enterococcus, Caulobacteraceae,* and *Lactobacillales*, have been described as being more enriched in bronchoalveolar lavage of subjects with lung adenocarcinoma^27^. We hypothesize that in thoracic malignancies, such as NSCLC and malignant pleural mesothelioma, these bacteria (or bacterial DNA) travel from the GI tract to the lungs through microaspiration, then translocate through disrupted lung–pleura barrier into the pleural space. Furthermore, the increased bacterial load of pleural fluid in mesothelioma may be related to direct malignant involvement and disruption of the pleural-peritoneal barrier, allowing for direct translocation of GI-specific taxa into the pleural space. The low oxygen content of the pleural space may have prevented additional GI commensals from surviving and being recovered by 16S rRNA gene sequencing. Previous studies have shown that paracellular permeability and tight junction barrier between the pleural mesothelial cell like play an important role in pleural fluid composition as well as cancer metastasis into the pleural space^28,29^. The pleural microbiome may be contributing to this increase permeability; alternatively, the increased permeability may allow for increased passage of organisms and microbial signals into the pleural space. Future investigations that pair pleural microbiome and host molecular expression patterns in human and murine models will be needed determine the exact mechanism and causality of these interactions.

On the other hand, pleural effusions from the MPE-Other, Paramalignant, and Benign groups were less likely to be enriched with these oral and GI taxa, probably because the lungs are not directly impacted to allow for translocation of microaspirated bacteria. Interestingly, we did not find GI-specific taxa enriched in pleural fluid of the MPE-Other group, which included metastatic malignancies of GI origin. This may be due to the heterogeneous nature of this group with many different types of primary malignancies that are in communication with different mucosal microbial environments.

Of note is the enrichment of *Deinococcus* in pleural fluid for the Paramalignant group. This bacterium has been described in several other malignancies, including premalignant lesions in gastric adenocarcinoma^30–32^. It is possible that enrichment with *Deinococcus* could be associated with subsequent development of frank MPE; a larger study would be needed to confirm this finding.

In our survival analysis, we used both LEfSe and random forest classifiers to identify taxa that are associated with mortality. The random forest classifiers identified promising AUCs, with some exceeding the thresholds commonly regarded as being of clinical us. However, given the lack of a validation set, we cannot conclude that we have developed a clinically useful biomarker. Nonetheless, subjects with early mortality in the MPE-Lung group had fluid enriched with *Cellvibrio*, whereas those with prolonged survival had fluid enriched with *Shewanella* and *Deinococcus*. This observation is in agreement with other investigations, which showed *Shewanella* to be more enriched in bronchoalveolar lavage of subjects without metastatic lung adenocarcinoma than those with metastatic disease^27^. *Bifidobacterium* was identified through random forest classifier for MPE-Lung as predictive of worse prognosis. This is contradictory to previous literature, where *Bifidobacterium* has been shown to be associated with decreased tumor burden and increased response to immune checkpoint inhibitors in NSCLC^33–35^. Thus, this taxa's contribution to the random forest classifier deserves further validation in subsequent investigations.

Late mortality in the MPE-Lung and Mesothelioma groups was associated with enrichment of *Deinococcus* in the pleural fluid. *Deinococcus* has been described in microbiota of other malignant and pre-malignant states; its association with prolonged survival in MPE-Lung and Mesothelioma is worth further investigation^31,32^.

Early mortality in subjects with MPE-Other and Mesothelioma had fluid enriched with *Enterococcus* and *Prevotella*, respectively. These are gut and oral commensals. We have previously shown that enrichment of the lower airway with oral taxa, including *Prevotella*, was associated with increased inflammatory tone, upregulation of host transcriptomic signatures associated with carcinogenesis, greater stage, and worse prognosis^16,22,36,37^.

The survival analysis suggest that microbial signatures associated with prognosis are primary site specific, probably reflective of the different mechanisms that lead to pleural involvement. Collection of tissue from the primary malignancy, in addition to the pleural fluid, would have been helpful to correlate this observation. In addition, it would be of interest to explore the metabolomic and transcriptomic landscape of the pleural space to further elucidate key pathways associated with early mortality.

This is a single center study, which is a limitation of our investigation. Due to this study’s cross-sectional design, many subjects did not have repeat samples to allow for longitudinal analysis. Another limitation of this work is that 16S rRNA gene sequencing is unable to reveal whether the identified bacteria are alive or dead; thus, signals described here may represent microbial DNA that has translocated into the pleural space. Even so, the presence of microbial products could still influence the host immune system and cancer progression. In future investigations, it will be important to include profiling of the host immune tone to explore further the nature of host-microbial interactions in malignant pleural effusions. Additionally, longitudinal samples may help to understand microbial dynamics and to evaluate treatment effects. Finally, pre-clinical murine models are needed to validate our findings and experimentally evaluate the nature of the associations identified in this human cohort.

In summary, we describe microbial genomic signatures present in pleural fluid of patients with malignancy associated with distinct diagnosis and prognosis. Further investigation of pleural microbiome and other components of this complex space may lead to the development biomarkers for diagnosis and prognosis. These investigations may ultimately uncover novel mechanisms of microbial/host cross talk in the pleural fluid that may be novel therapeutic targets.

## Methods

### Subject recruitment and sample collection

Adult subjects who were planned to undergo any clinically indicated procedures for pleural fluid sampling at NYU Langone Medical Center were prospectively recruited between February 1999 and January 2021. These procedures included thoracentesis, indwelling pleural catheter placement, medical pleuroscopy, video-assisted thoracoscopic surgery, and open thoracotomy. Subjects with evidence of an infected pleural space or small cell lung cancer were excluded from analysis. Written informed consent was obtained from all patients prior to enrollment in the study. This study was approved by the New York University Institution Review Board (IRB# s16-01598). All experiments were performed in accordance with relevant named guidelines and regulations.

The procedures for pleural fluid collection were performed under sterile conditions in the standard fashion by experienced physicians. After an adequate volume of fluid was collected and sent for clinically-indicated tests, the remaining fluid was sterilely collected in test tubes with DNA-stabilizing solution and stored in secured freezers at − 80 °C. Corresponding background samples were collected prior to the procedure by swabbing sterile surgical equipment. Corresponding skin samples were also collected by swabbing the patient’s skin after preparation with antiseptic solution, per procedural protocol. These background and skin swab samples were collected in test tubes and stored in secured freezers at − 80 °C.

The subjects’ demographic and clinical information were collected by review of the electronic medical record. Periodic follow-up of these subjects was performed at predetermined intervals according to the IRB protocol and at time of specimen processing. At the time of specimen processing, the samples were thawed and tests were performed by blinded laboratory personnel. A timeline for each participant’s enrollment, sample collection date, and duration of follow-up is shown in Supplemental Fig. S5.

### Subject demographics, sample characteristics, and definitions of groups

A total of 168 pleural effusion samples were collected from 168 unique subjects. One subject had evidence of an infected pleural space with positive pleural fluid bacterial cultures; two subjects were diagnosed with malignant pleural effusions related to small cell lung cancer. These three subjects and their corresponding samples were excluded from further analysis. The remaining 165 pleural fluid samples from 165 unique subjects were included for analysis; their demographics can be found in Table 1. These samples were divided into five groups based on histopathologic definitions: Benign (*n* = 16), Paramalignant (*n* = 21), MPE-Lung (*n* = 57), MPE-Other (*n* = 22), and Mesothelioma (*n* = 49) groups.

The Benign group was defined as pleural fluid without cytologic or pathologic evidence of malignancy and were collected from subjects without active malignancy. Active malignancy was defined as cancer diagnosed within the previous six months, cancer treated within six months, or cancer that is not yet in complete remission. On subsequent follow-up (median 21.4 [IQR 5.8, 33.8] months), none of these subjects were found to have malignancy. Characteristics and etiologies of Benign group pleural effusions can be found in Supplemental Table S1.

The Paramalignant group was defined as pleural effusions in subjects with active malignancy, but without cytologic or pathologic evidence of malignant involvement of the pleural space. All 21 samples had fluid cytology negative for malignancy; four of 21 also had pleural biopsies performed that were negative for malignancy. On subsequent follow-up (median 20.9 [IQR 12, 33] months), all subjects had repeat pleural fluid sampling at least once and none developed cytologic or pathologic evidence of MPE. The pathology and sites of primary malignancies can be found in Supplemental Table S2.

The MPE-Lung group was defined as MPE in the setting of an active malignancy of lung origin (i.e., NSCLC). Specifically, all had fluid cytology that was positive for malignant cells of lung origin; pleural biopsies, if performed, were also positive for malignancy of lung origin. The MPE-Other group was defined as MPE in the setting of an active malignancy of extra-thoracic origin. All in this group had pleural fluid cytology positive for malignant cells of extra-thoracic origin. The Mesothelioma group was defined as malignant pleural effusions related to malignant pleural mesothelioma; all subjects in this group had pleural biopsies demonstrating malignant pleural mesothelioma. Pathology and sites of primary malignancy for MPE can be found in Supplemental Table S2.

In addition to pleural fluid samples, a total of 58 background samples (swabs of sterile/surgical equipment, reagent control samples, and mock mixed microbial DNA) and 21 skin samples (collected immediately after preparation with antiseptic solution, per procedural protocol) were also collected.

### Bacterial 16S rRNA-encoding gene sequencing and bacterial load assessment

High-throughput sequencing of bacterial 16S rRNA-encoding gene amplicons (V4 region^38^) was performed on pleural fluid samples. Reagent control samples, mock mixed microbial DNA, background, and skin samples were also sequenced and analyzed in parallel. The obtained 16S rRNA gene sequences were analyzed and had taxonomy assigned with the Quantitative Insights into Microbial Ecology 2 (QIIME2) package (version April 2021). ASV (amplicon sequence variant) were not removed from upstream analysis.

Bacterial load was measured in background, skin, and pleural fluid samples by a droplet digital PCR system (ddPCR), as described elsewhere^39^.

### Quality control and identification of potential bacterial contaminants

Median sequence depth was 21,138 [IQR 12,489, 29,502] per sample; sequencing depth was comparable between groups with few outliers (Supplemental Fig. S1). All samples had read depths of at least 1000. There was a total of 11,250 taxa identified. After removal of taxa with zero abundance among all samples and agglomerating at the genus-level, there were 862 taxa remaining. Taxa that were not seen more than 5 times in at least 1% of samples were filtered, resulting in 551 taxa for downstream analysis.

To account for low biomass of samples and potential contaminants, a prevalence-based method from the *R* package *decontam* (version 1.14.0) was used to identify potential bacterial contaminants from the sequencing datasets. In this process, all reads from pooled pleural fluid samples were compared against background controls and skin swabs. For the analyses presented here, all potential contaminants were identified as described. A total of 428 of 539 were identified as potential contaminants; although these taxa were not removed from subsequent analyses, their label as potential contaminant was kept. A complete list of potential contaminants ranked by relative abundance in each sample type is available in Supplemental Fig. S7 and Supplemental Table S10.

### Assessment of diversity and differential enrichment

Microbiome diversity indices were evaluated by comparing alpha diversity by Shannon diversity indices and beta diversity principal coordinate analysis (PCoA) based on Bray–Curtis dissimilarity index. Differential enrichment analysis with linear discriminant analysis effect size (LEfSe) using Kruskal–Wallis rank sum test cutoff of 0.05, Wilcoxon rank sum test cutoff of 0.05, and linear discriminate analysis (LDA) score cutoff of 2. Random forest classifiers were performed using 5-k fold cross validation. The above analyses and Dirichlet multinominal mixtures (DMM) modeling were performed in *R* version 4.1.2.

## Supplementary Information

Supplementary Information.

Supplementary Tables.

## Data Availability

Data from the 16S rRNA gene sequencing is available at BioProject ID PRJNA865295. *R* scripts used for analyses are available at https://github.com/segalmicrobiomelab/malignant_pleural_fluid.
