# Supplementary material for: Pleural fluid microbiota as a biomarker for malignancy and prognosis
Source: Sci Rep. 2023 Feb 8;13:2229. doi: 10.1038/s41598-023-29001-4 (PMC9908925; doi:10.1038/s41598-023-29001-4)
Supplement: Supplementary file 1 — Supplementary Information. [file 41598_2023_29001_MOESM1_ESM.pdf]

Supplemental Information File

TITLE: **Pleural fluid microbiota as a biomarker for malignancy and prognosis**

*Benjamin Kwok[1], Benjamin G. Wu[1,2], Ibrahim F. Kocak[1], Imran Sulaiman[1,3,4], Rosemary Schluger[1], Yonghua Li[1], Raheel Anwer[1], Chandra Goparaju[5], Daniel J. Ryan[3,4], Marla Sagatelian[6], Matthew S. Dreier[7], Vivek Murthy[1,5], Samaan Rafeq[1,5], Gaetane C. Michaud[8], Daniel H. Sterman[1,5], Jamie L. Bessich[1,5], Harvey I. Pass[5], Leopoldo N. Segal[1], Jun-Chieh J. Tsay[1,2]**

[1] Division of Pulmonary, Critical Care, and Sleep Medicine, New York University Grossman School of Medicine, New York, NY, USA
[2] Division of Pulmonary and Critical Care Medicine, Veterans Affairs New York Harbor Healthcare System, New York, NY, USA
[3] Department of Respiratory Medicine, Royal College of Surgeons in Ireland, Dublin, Ireland
[4] Department of Respiratory Medicine, Beaumont Hospital, Dublin, Ireland
[5] Department of Cardiothoracic Surgery, New York University Grossman School of Medicine, New York, NY, USA
[6] School of Medicine, Medical University of South Carolina, Charleston, SC, USA
[7] New York University Grossman School of Medicine, New York, NY, USA
[8] Division of Pulmonary, Critical Care, and Sleep Medicine, University of South Florida Health, Tampa, FL, USA

Benjamin Kwok, MD	Benjamin.Kwok@nyulangone.org
Benjamin G. Wu, MD	Benjamin.Wu@nyulangone.org
Ibrahim F. Kocak, MD	Ibrahim.Kocack@nyulangone.org
Imran Sulaiman, MD, PhD	SheikMohammadImran.Sulaiman@nyulangone.org
Rosemary Schluger, RN	Rosemary.Schluger@nyulangone.org
Yonghua Li, MD, PhD	Yonghua.Li@nyulangone.org
Raheel Anwer	Raheel.Anwer@nyulangone.org
Chandra Goparaju, PhD	Chandra.Goparaju@nyulangone.org
Daniel J. Ryan, MD	danieljohnryan@beaumont.ie
Marla Sagatelian	sagateli@musc.edu
Matthew S. Dreier	Matthew.Dreier@nyulangone.org
Vivek Murthy, MD	Vivek.Murthy@nyulangone.org
Samaan Rafeq, MD	Samaan.Rafeq@nyulangone.org
Gaetane C. Michaud, MD	GaetaneMichaud@usf.edu
Daniel H. Sterman, MD	Daniel.Sterman@nyulangone.org
Jamie L. Bessich, MD	Jamie.Bessich@nyulangone.org
Harvey I. Pass, MD	Harvey.Pass@nyulangone.org
Leopoldo N. Segal, MD	Leopoldo.Segal@nyulangone.org
Jun-Chieh J. Tsay, MD	Jun-chieh.Tsay@nyulangone.org

*Corresponding author/address for reprints:
Jun-Chieh J. Tsay, MD Jun-chieh.Tsay@nyulangone.org

NYU School of Medicine
462 First Avenue 7N21
New York, NY 10016
Tel: (212) 263-6479
Fax: (212) 263-8441

Supplemental Table S1. Characteristics of Benign Pleural Effusions

Characteristics	Benign Pleural Fluid N = 16[a]
**Fluid chemistry**	
Fluid protein (grams/deciliter)	3.6 [2.65, 4.35]
Fluid LDH[b] (units/liter)	138 [106, 176]
**Classification[c]**	
Transudate	8 (50%)
Exudate	8 (50%)
**Etiology[d]**	
Heart failure	6 (37.5%)
Hepatic hydrothorax	3 (18.75%)
Chronic kidney disease	7 (43.75%)
Chronic pleuritis	6 (37.5%)
Connective tissue disease	6 (37.5%)
Post-operative	3 (18.75%)
Thoracic endometriosis	1 (6.25%)
ATTR amyloidosis	1 (6.25%)
Infection	0 (0%)

[a]Median (IQR); n (%)
[b]LDH, lactate dehydrogenase. Upper limit of normal is 220 units/liter.
[c]Light's criteria for pleural fluid.
[d]Effusions with more than one etiology are listed multiple times.

Supplemental Table S2. Characteristics of Paramalignant and Malignant Pleural Effusions

Characteristic	Overall N = 149[a]	Paramalignant N = 21[a]	MPE-Lung N = 57[a]	MPE-Other N = 22[a]	Mesothelioma N = 49[a]	p-value[b]
**Pathology**						
Adenocarcinoma	66 (44%)	5 (24%)	45 (79%)	16 (73%)	0 (0%)	<0.05[c,d]
Squamous	9 (6.0%)	4 (19%)	4 (7.0%)	1 (4.5%)	0 (0%)	0.165
NSCLC nos	18 (12%)	10 (48%)	8 (14%)	0 (0%)	0 (0%)	0.004[c]
Hematologic	6 (4.0%)	2 (9.5%)	0 (0%)	4 (18%)	0 (0%)	0.482
Renal cell	1 (0.7%)	0 (0%)	0 (0%)	1 (4.5%)	0 (0%)	
Mesothelioma						
Epithelial	43 (29%)	0 (0%)	0 (0%)	0 (0%)	43 (88%)	
Biphasic	4 (2.7%)	0 (0%)	0 (0%)	0 (0%)	4 (8.2%)	
Sarcomatoid	2 (1.3%)	0 (0%)	0 (0%)	0 (0%)	2 (4.1%)	
**Primary malignancy**						
Breast	4 (2.7%)	0 (0%)	0 (0%)	4 (18%)	0 (0%)	
Gastrointestinal	8 (5.4%)	0 (0%)	0 (0%)	8 (36%)	0 (0%)	
Genitourinary	2 (1.3%)	0 (0%)	0 (0%)	2 (9.1%)	0 (0%)	
Gynecological	4 (2.7%)	0 (0%)	0 (0%)	4 (18%)	0 (0%)	
Hematologic	6 (4.0%)	2 (9.5%)	0 (0%)	4 (18%)	0 (0%)	
Lung	76 (51%)	19 (90%)	57 (100%)	0 (0%)	0 (0%)	*ns*
Mesothelioma	49 (33%)	0 (0%)	0 (0%)	0 (0%)	49 (100%)	

NSCLC nos, non-small cell lung cancer not otherwise specified. *ns*, not significant

[a]n (%)

[b]Kruskal-Wallis rank sum test; Pearson's Chi-squared test; Wilcox rank sum test.

[c]Chi-square test between groups Paramalignant and MPE-Lung had a p-value <0.05.

[d]Chi-square test between groups Paramalignant and MPE-Other had a p-value <0.01.

Supplemental Table S4. Demographics of subjects stratified by DMM cluster

		DMM Clusters			
**Characteristic**	**Overall, N = 165**[1]	**Cluster 1, N = 81**[1]	**Cluster 2, N = 581**	**Cluster 3, N = 261**	**p-value**[2]
**Age**	71 (62, 79)	72 (62, 81)	71 (62, 78)	66 (59, 75)	0.3
**Gender (male)**	83 (50%)	34 (42%)	31 (53%)	18 (69%)	0.045
**BMI**	24.9 (22.3, 27.5)	25.1 (23.7, 28.0)	23.9 (21.1, 26.9)	25.0 (21.9, 27.2)	0.089
**Race**					0.063
Caucasian	122 (74%)	51 (63%)	46 (79%)	25 (96%)	
Hispanic	5 (3.0%)	3 (3.7%)	1 (1.7%)	1 (3.8%)	
African-American	11 (6.7%)	7 (8.6%)	4 (6.9%)	0 (0%)	
Asian	11 (6.7%)	8 (9.9%)	3 (5.2%)	0 (0%)	
Other	16 (9.7%)	12 (15%)	4 (6.9%)	0 (0%)	
**Smoker**	92 (56%)	45 (56%)	34 (59%)	13 (50%)	0.8
Pack-years	2 (0, 24)	2 (0, 30)	5 (0, 25)	1 (0, 19)	0.9
**Asbestos exposure**	33 (20%)	2 (2.5%)	15 (26%)	16 (62%)	<0.001
**Comorbidities**					
Hyperlipidemia	74 (45%)	36 (44%)	28 (48%)	10 (38%)	0.7
Hypertension	85 (52%)	44 (54%)	31 (53%)	10 (38%)	0.3
Heart failure	24 (15%)	16 (20%)	3 (5.2%)	5 (19%)	0.033
CAD	33 (20%)	14 (17%)	12 (21%)	7 (27%)	0.6
CVA	9 (5.5%)	4 (4.9%)	5 (8.6%)	0 (0%)	0.4
Diabetes mellitus	31 (19%)	15 (19%)	10 (17%)	6 (23%)	0.8
Asthma	8 (4.8%)	4 (4.9%)	2 (3.4%)	2 (7.7%)	0.7
COPD	25 (15%)	14 (17%)	10 (17%)	1 (3.8%)	0.2
CTD	15 (9.1%)	8 (9.9%)	6 (10%)	1 (3.8%)	0.7
CKD	17 (10%)	13 (16%)	3 (5.2%)	1 (3.8%)	0.072
Cirrhosis	4 (2.4%)	3 (3.7%)	1 (1.7%)	0 (0%)	0.8
HIV infection	0 (0%)	0 (0%)	0 (0%)	0 (0%)	
GERD	49 (30%)	26 (32%)	14 (24%)	9 (35%)	0.5
**Pleural fluid groups**					
Benign	16	13 (16%)	3 (5.2%)	0 (0%)	<0.001
Paramalignant	21	15 (19%)	4 (6.9%)	2 (7.7%)	<0.001
MPE-Lung	57	32 (40%)	25 (43%)	0 (0%)	<0.001
MPE-Other	22	19 (23%)	3 (5.2%)	0 (0%)	<0.001
Mesothelioma	49	2 (2.5%)	23 (40%)	24 (92%)	<0.001

[1]Median (IQR); n (%)

[2]Kruskal-Wallis rank sum test; Wilconxon rank sum test; Pearson's Chi-squared test with Bonoferroni adjustment for post-hoc analysis; Fisher's exact test.

NSCLC nos, non-small cell lung cancer not otherwise specified. CAD, coronary artery disease. CVA, cerebrovascular accident. COPD, chronic obstructive pulmonary disease. CTD, connective tissue disease. CKD, chronic kidney disease. HIV, human immunodeficiency disease infection. GERD, gastroesophageal reflux disease.

Supplemental Table S5. Demographics of subjects with MPE-Lung, MPE-Other, and Mesothelioma stratified by DMM Cluster

	MPE-Lung			MPE-Other			Mesothelioma			
	DMM Clusters			DMM Clusters			DMM Clusters			
**Characteristic**	**Cluster 1, N = 321**	**Cluster 2, N = 251**	***p*-value[2]**	**Cluster 1, N = 191**	**Cluster 2, N = 31**	***p*-value[2]**	**Cluster 1, N = 21**	**Cluster 2, N = 231**	**Cluster 3, N = 241**	***p*-value[2]**
**Age**	68 (61, 77)	75 (62, 80)	0.4	74 (66, 82)	51 (49, 58)	0.035	61 (61, 61)	70 (62, 76)	66 (59, 74)	0.3
**Gender (male)**	13 (41%)	10 (40%)	>0.9	7 (37%)	1 (33%)	>0.9	1 (50%)	17 (74%)	16 (67%)	0.7
**BMI**	24.8 (23.1, 27.2)	23.3 (21.3, 26.8)	0.2	25 (23, 30)	21 (20, 24)	0.4	26 (26, 26)	24 (22, 28)	25 (22, 27)	0.9
**Race**			0.12			0.2				0.004
Caucasian	15 (47%)	20 (80%)		14 (74%)	1 (33%)		0 (0%)	19 (83%)	23 (96%)	
Hispanic	2 (6.2%)	0 (0%)		0 (0%)	0 (0%)		0 (0%)	1 (4.3%)	1 (4.2%)	
African-American	2 (6.2%)	1 (4.0%)		3 (16%)	1 (33%)		0 (0%)	2 (8.7%)	0 (0%)	
Asian	8 (25%)	2 (8.0%)		0 (0%)	0 (0%)		0 (0%)	0 (0%)	0 (0%)	
Other	5 (16%)	2 (8.0%)		2 (11%)	1 (33%)		2 (100%)	1 (4.3%)	0 (0%)	
**Smoker**	20 (62%)	16 (64%)	>0.9	8 (42%)	1 (33%)	>0.9	1 (50%)	12 (52%)	11 (46%)	0.9
Pack-years	5 (0, 34)	5 (0, 32)	>0.9	0 (0, 5)	0 (0, 0)	0.7	6 (3, 8)	0 (0, 15)	0 (0, 16)	>0.9
**Asbestos exposure**	0 (0%)	1 (4.0%)	0.4	0 (0%)	0 (0%)		1 (50%)	14 (61%)	16 (67%)	0.9
**Comorbidities**										
Hyperlipidemia	12 (38%)	7 (28%)	0.5	10 (53%)	0 (0%)	0.2	1 (50%)	15 (65%)	9 (38%)	0.11
Hypertension	15 (47%)	10 (40%)	0.6	13 (68%)	2 (67%)	>0.9	0 (0%)	14 (61%)	9 (38%)	0.094
Heart failure	4 (12%)	2 (8.0%)	0.7	2 (11%)	0 (0%)	>0.9	0 (0%)	1 (4.3%)	3 (12%)	0.7
CAD	7 (22%)	4 (16%)	0.7	2 (11%)	0 (0%)	>0.9	0 (0%)	5 (22%)	6 (25%)	>0.9
CVA	2 (6.2%)	3 (12%)	0.6	0 (0%)	0 (0%)		0 (0%)	1 (4.3%)	0 (0%)	0.5
Diabetes mellitus	5 (16%)	3 (12%)	>0.9	4 (21%)	0 (0%)	>0.9	0 (0%)	5 (22%)	6 (25%)	>0.9
Asthma	2 (6.2%)	0 (0%)	0.5	2 (11%)	0 (0%)	>0.9	0 (0%)	2 (8.7%)	2 (8.3%)	>0.9
COPD	6 (19%)	2 (8.0%)	0.4	3 (16%)	0 (0%)	>0.9	0 (0%)	5 (22%)	0 (0%)	0.047
CTD	0 (0%)	2 (8.0%)	0.2	2 (11%)	0 (0%)		0 (0%)	2 (8.7%)	1 (4.2%)	0.7
CKD	3 (9.4%)	1 (4.0%)	0.6	2 (11%)	0 (0%)	>0.9	0 (0%)	2 (8.7%)	1 (4.2%)	0.7
Cirrhosis	0 (0%)	0 (0%)		0 (0%)	0 (0%)		0 (0%)	1 (4.3%)	0 (0%)	0.5
HIV infection	0 (0%)	0 (0%)		0 (0%)	0 (0%)		0 (0%)	0 (0%)		
GERD	10 (31%)	6 (24%)	0.5	7 (37%)	2 (67%)	0.5	0 (0%)	5 (22%)	8 (33%)	0.6
**Pathology**			0.019			>0.9				0.2
Adenocarcinoma	29 (91%)	16 (64%)		13 (68%)	3 (100%)		-	-	-	
Squamous	2 (6.2%)	2 (8.0%)		1 (5.3%)	0 (0%)		-	-	-	
NSCLC, nos	1 (3.1%)	7 (28%)		-	-		-	-	-	
Hematologic	-	-		4 (21%)	0 (0%)		-	-	-	
Renal cell	-	-		1 (5.3%)	0 (0%)		-	-	-	
Mesothelioma	-	-		-	-		-	-	-	
Epithelial	-	-		-	-		2 (100%)	21 (91%)	20 (83.4%)	
Biphasic	-	-		-	-		0 (0%)	2 (8.7%)	2 (8.3%)	
Sarcomatoid	-	-		-	-		0 (0%)	0 (0%)	2 (8.3%)	
**Site of primary malignancy**			ns			0.3				ns
Breast	-	-		4 (21%)	0 (0%)		-	-	-	
Gastrointestinal	-	-		7 (37%)	1 (33%)		-	-	-	
Genitoureteral and renal	-	-		2 (11%)	0 (0%)		-	-	-	
Gynecological	-	-		2 (11%)	2 (67%)		-	-	-	
Lung	32 (100%)	25 (100%)		0 (0%)	0 (0%)		-	-	-	
Hematologic	-	-		4 (21%)	0 (0%)		-	-	-	
Mesothelioma	-	-		-	-		2 (100%)	23 (100%)	23 (100%)	

[1]Median (IQR); n (%) - -

[2]Wilcoxon rank sum test; Pearson's Chi-squared test; Fisher's exact test

NSCLC nos, non-small cell lung cancer not otherwise specified. CAD, coronary artery disease. CVA, cerebrovascular accident. COPD, chronic obstructive pulmonary disease. CTD, connective tissue disease. CKD, chronic kidney disease. HIV, human immunodeficiency disease infection. GERD, gastroesophageal reflux disease. ns, not significant.

Supplemental Figure S1.

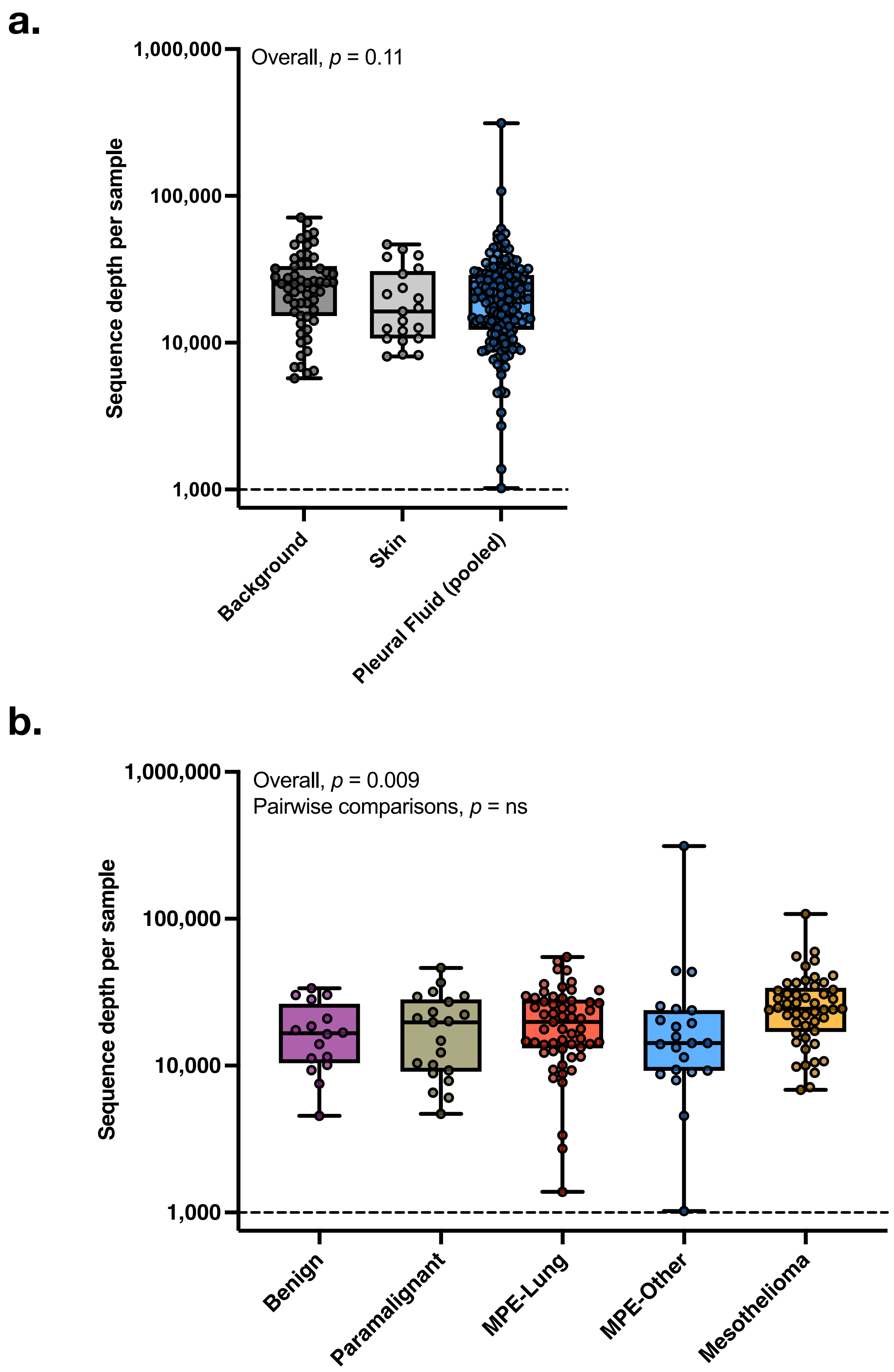

Supplemental Figure S1. Sequence depth for each type of pleural fluid sample. a. Comparison of background, skin, and pooled pleural fluid samples. **b.** Comparison of different groups of pleural fluid samples. Overall *p*-value by Kruskal-Wallis rank sum test. Pairwise comparisons by Wilcoxon rank sum tests with Benjamini-Hochberg adjustment for multiple comparisons. ns, not significant.

Supplemental Figure S2.

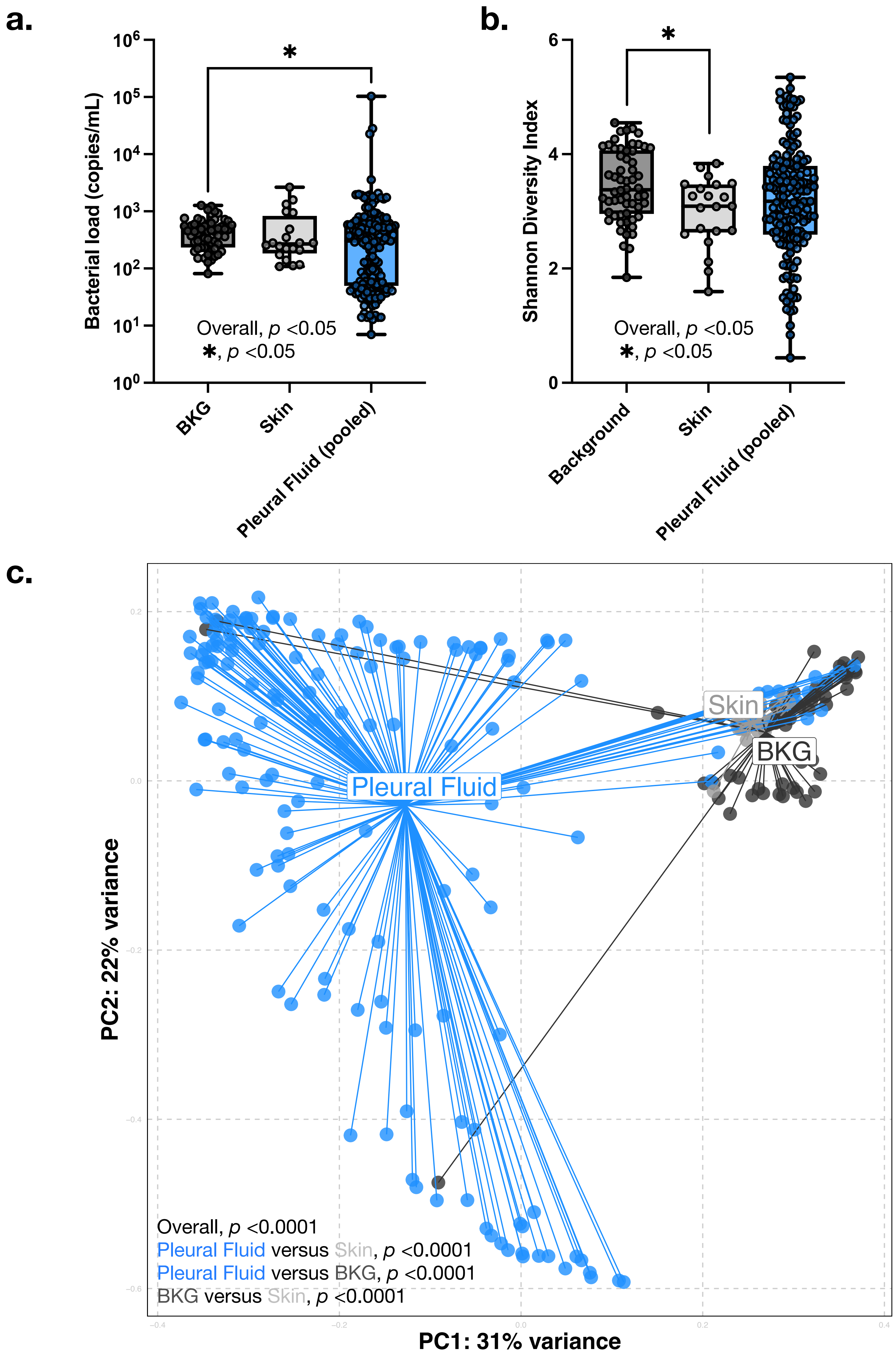

Supplemental Figure S2. Topographic analyses of pooled pleural fluid samples compared against background and skin samples. a. Comparison of bacterial load, as measured by ddPCR. p-values by Kruskal-Wallis rank sum test; individual comparisons by Wilcoxon rank sum test with Benjamini-Hochberg adjustment for multiple comparisons. b. Comparison of alpha diversities (Shannon diversity) of each group. p-values by Kruskal-Wallis rank sum test; individual comparisons by Wilcoxon rank sum tests with Benjamini-Hochberg adjustment for multiple comparisons. c. Comparison of beta diversity (Bray-Curtis dissimilarity index) for each group. p-values by PERMANOVA.

Supplemental Figure S3.

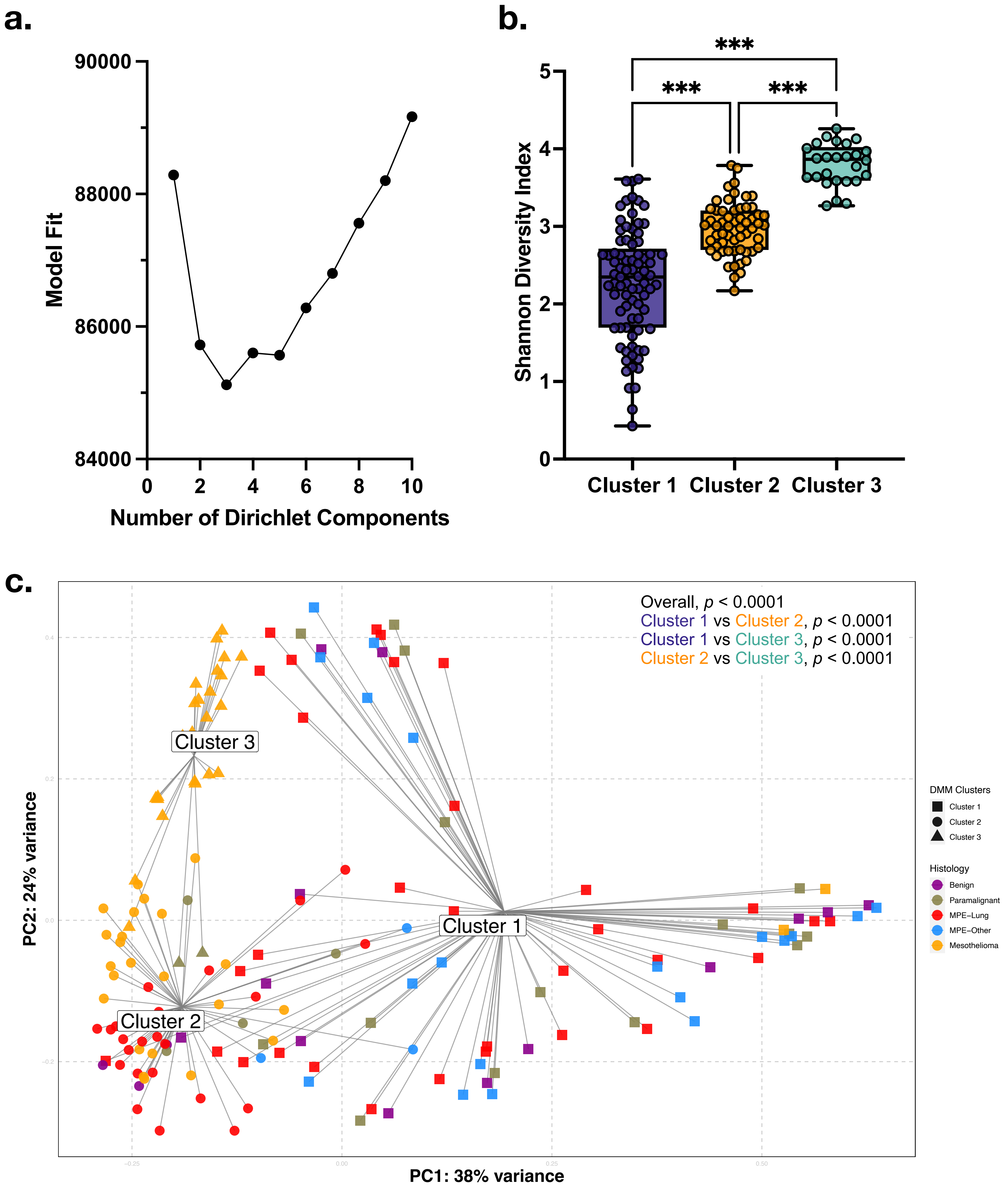

Supplemental Figure S3. Dirichlet multinominal mixtures model (DMM) fit of pleural fluid samples. a. LaPlace approximation of DMM model fit indicate that three distinct pleural fluid microbiota clusters are present in our cohort. **b.** Alpha (Shannon) diversity. *p*-values by Kruskal-Wallis rank sum test. Individual comparisons by Wilcoxon rank sum tests with Benjamini-Hochberg adjustment for multiple comparisons. Overall *p*-value <0.0001, ***, p<0.0001. **c.** Beta diversity (Bray-Curtis) of these three clusters. *p*-values by PERMANOVA.

Supplemental Figure S4.

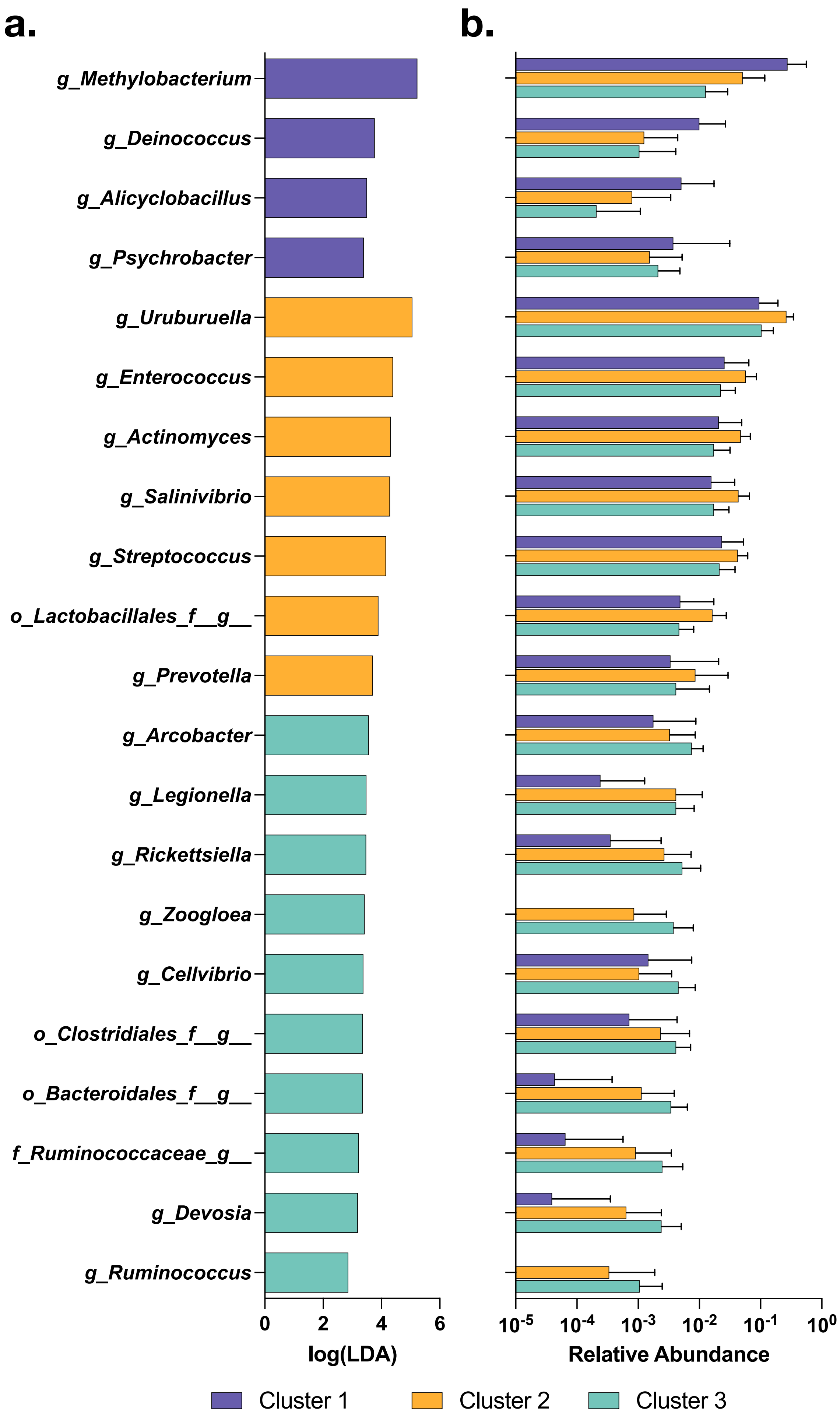

Supplemental Figure S4. Top taxa in each Dirichlet multinominal mixtures cluster by LEfSe. a. Taxa are ranked in decreasing order of linear discriminant analysis (LDA) score. **b.** Mean relative abundance for each taxa identified as enriched by LEfSe.

Supplemental Figure S5.

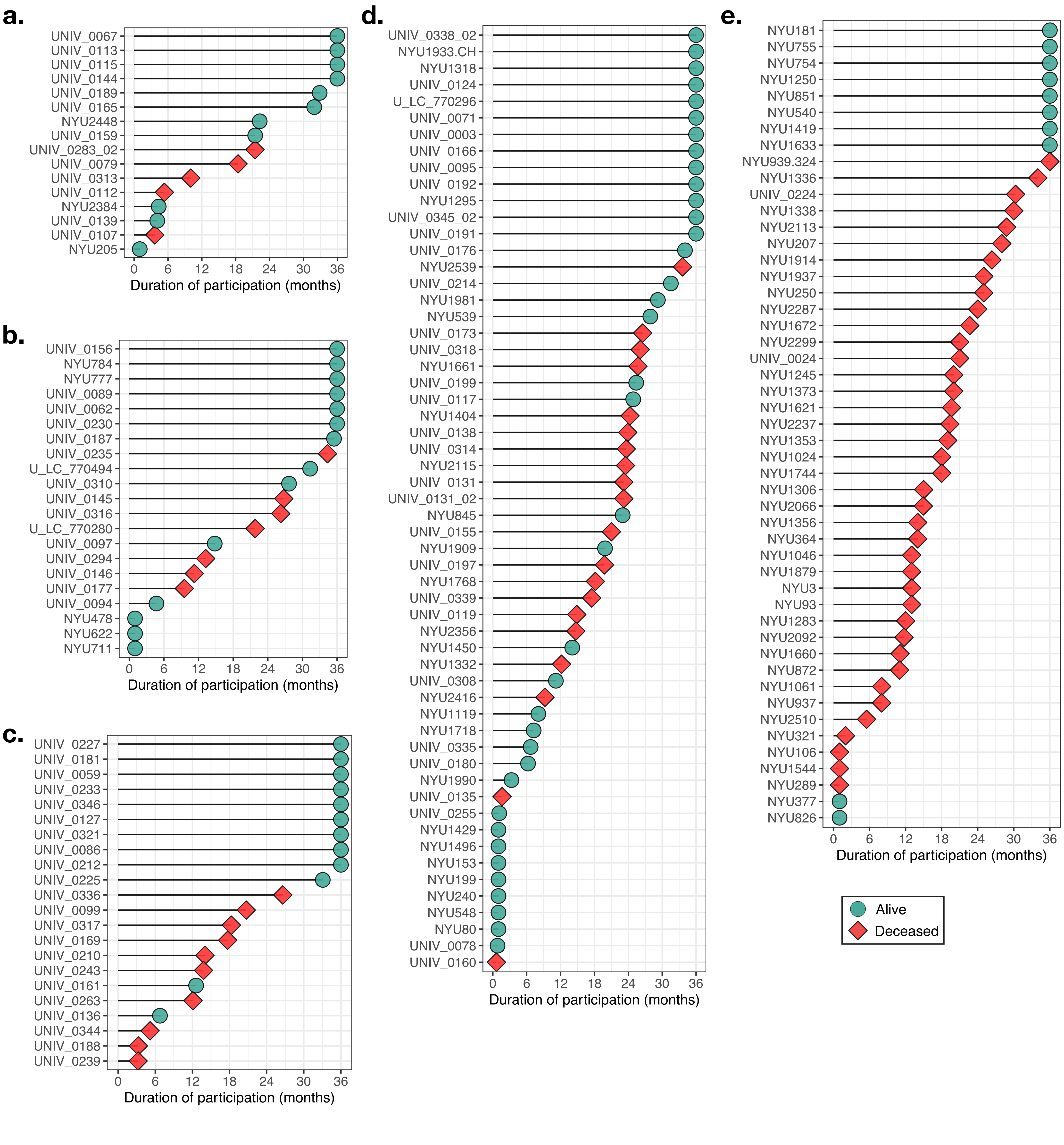

Supplemental Figure S5. Timeline of patient enrollment, sample collection, and duration of follow-up. Subjects in Benign (**a**), Paramalignant (**b**), MPE-Other (**c**), MPE-Lung (**d**), and Mesothelioma (**e**) groups. Date of enrollment into the study is also the date of specimen collection, which is taken to be time = 0 months. Subjects were followed longitudinally by scheduled phone calls and chart review at 6 month intervals and/or at the end of follow-up. Follow-up was stopped at 36 months or if subject was deceased, withdrew consent from the study, and lost-to-follow-up.

Supplemental Figure S6.

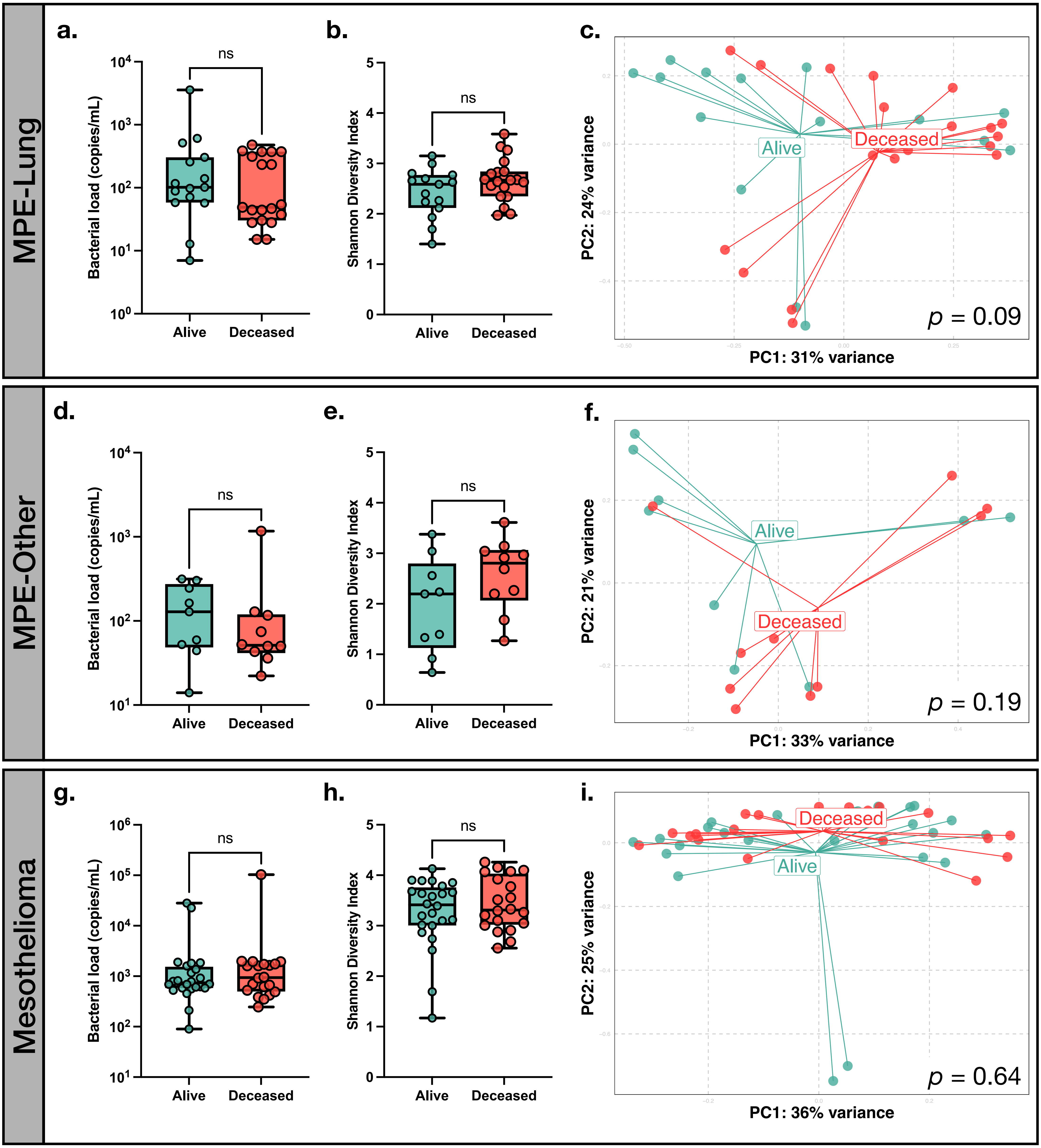

Supplemental Figure S6. Compositional differences in early and late mortality in malignant pleural effusions. a–c. Bacterial load, alpha diversity (Shannon diversity), and beta diversity (Bray-Curis dissimilarity index) for survival of subjects in the MPE-Lung group at time of median survival (33.6 months). **d–f.** Bacterial load, alpha diversity (Shannon diversity), and beta diversity (Bray-Curis dissimilarity index) for survival of subjects in the MPE-Other group at time of median survival (36 months). **g–i.** Bacterial load, alpha diversity (Shannon diversity), and beta diversity (Bray-Curis dissimilarity index) for survival of subjects in the Mesothelioma group at time of median survival (19.6 months). *p*-values by Wilcoxon rank sum test with Benjamini-Hochberg adjustment for multiple comparisons for bacterial concentration and alpha diversity. *p*-values by PERMANOVA for beta diversity. ns, not significant.

Supplemental Figure S7.

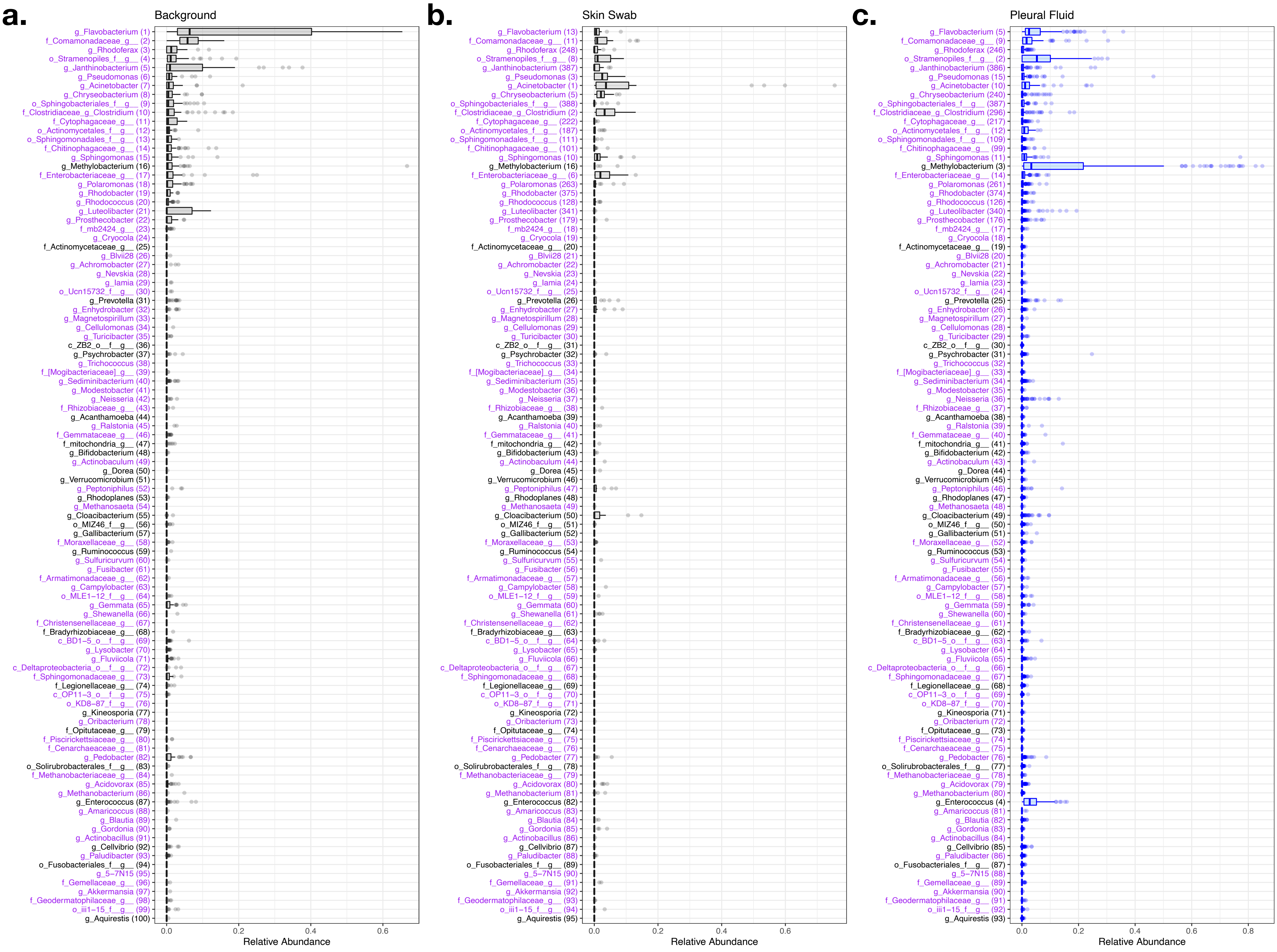

Supplemental Figure S7. Identification of potential contaminants in the top 100 taxa in background samples as compared with skin and pleural fluid samples. Boxplots showing the logarithmic relative abundance values of taxa rank ordered by dominance in background samples. The numbers in parentheses next to the taxa labels display the ranking in relative abundance for background, skin, or pleural fluid samples. Purple labels indicate taxa that are identified as potential contaminants by using a prevalence-based method.
